# Monitoring prevention or emergence of HIV drug resistance: results of a population-based foundational survey of early warning indicators in mainland Tanzania

**DOI:** 10.1186/1471-2334-14-196

**Published:** 2014-04-11

**Authors:** James M Juma, Jenny K Tiberio, Mathias I Abuya, Bonita K Kilama, Geoffrey R Somi, Veryeh Sambu, Richard Banda, Boniphace S Jullu, Angela A Ramadhani

**Affiliations:** 1 Ministry of Health and Social Welfare, The National AIDS Control Programme (NACP), P.O. Box 11857, Dar es Salaam, Tanzania; 2 University of California, San Francisco, Global Health Sciences, 50 Beale Street, Suite 1200, San Francisco, CA 94105-1823, USA; 3 World Health Organization, Tanzania Country Office, P.O. Box 9292, Dar es Salaam, Tanzania; 4 Afya Bora Consortium Global Health Leadership Fellowship Program (field attached fellow), University of Washington, 325 Ninth Avenue, Box 359909, Seattle, WA 98104-2499, USA; 5 St. Francis University College of Health and Allied Sciences, P.O. Box 175, Ifakara Morogoro, Tanzania

**Keywords:** Tanzania, HIV and AIDS, Care and treatment, Antiretroviral therapy (ART), Prevention, Emergence, Monitoring, HIV drug resistance (HIVDR), Early warning indicators

## Abstract

**Background:**

In Tanzania, routine individual-level testing for HIV drug resistance (HIVDR) using laboratory genotyping and phenotyping is not feasible due to resource constraints. To monitor the prevention or emergence of HIVDR at a population level, WHO developed generic strategies to be adapted by countries, which include a set of early warning indicators (EWIs).

**Methods:**

To establish a baseline of EWIs, we conducted a retrospective longitudinal survey of 35 purposively sampled care and treatment clinics in 17 regions of mainland Tanzania. We extracted data relevant for four EWIs (ART prescribing practices, patients lost to follow-up 12 months after ART initiation, retention on first-line ART at 12 months, and ART clinic appointment keeping in the first 12 months) from the patient monitoring system on patients who initiated ART at each respective facility in 2010. We uploaded patient information into WHO HIVResNet excel-based tool to compute national and facility averages of the EWIs and tested for associations between various programmatic factors and EWI performance using Fisher’s Exact Test.

**Results:**

All sampled facilities met the WHO EWI target (100%) for ART prescribing practices. However, the national averages for patients lost to follow-up 12 months after ART initiation, retention on first-line ART at 12 months, and ART clinic appointment keeping in the first 12 months fell short, at 26%, 54% and 38%, respectively, compared to the WHO targets ≤ 20%, ≥ 70%, and ≥ 80%. Clinics with fewer patients lost to follow-up 12 months after ART initiation and more patients retained on first-line-ART at 12 months were more likely to have their patients spend the longest time in the facility (including wait-time and time with providers), (p = 0.011 and 0.007, respectively).

**Conclusion:**

Tanzania performed very well in EWI 1a, ART prescribing practices. However, its performance in other three EWIs was far below the WHO targets. This study provides a baseline for future monitoring of EWIs in Tanzania and highlights areas for improvement in the management of ART patients in order not only to prevent emergence of HIVDR due to programmatic factors, but also to improve the quality of life for ART patients.

## Background

Antiretroviral therapy (ART) extends the lives of persons living with HIV and AIDS and improves the quality of their lives [1,2]. The rapid increase in ART coverage in low- and middle-income countries, especially in Sub-Saharan Africa, has also led to a clear decline in premature mortality [3,4]. ART, however, is not without complications, and suboptimal adherence can lead to therapeutic failure and the development of antiretroviral resistance [2]. The development of resistance requires changes in ART regimens with limited therapeutic choices each time resistance occurs. Additionally, individuals with drug-resistant HIV strains can transmit them to infants or sexual and needle-sharing partners, leading to infections that are already resistant to first-line therapy and more difficult to treat [2].

In Tanzania, like most low-income country settings, routine individual-level testing for HIV drug resistance (HIVDR) using laboratory genotyping and phenotyping is not feasible due to resource constraints [5,6]. To monitor the appearance of HIVDR at the population level, the World Health Organization (WHO) developed a generic protocol for each country to adapt based on the routine patient monitoring system (PMS) in order to monitor and mitigate HIVDR [5]. The protocol focuses on eight elements: (i) creating an HIVDR technical working group to develop and implement the strategy, (ii) monitoring HIVDR early warning indicators (EWIs) at ART sites, (iii) conducting sentinel monitoring of HIVDR during treatment, (iv) conducting HIVDR threshold surveys to assess the incidence of transmitted HIVDR, (v) establishing an HIVDR database, (vi) designating an in-country or regional WHO-accredited HIVDR genotyping laboratory, (vii) conducting a national-level annual HIVDR situation review and making recommendations and (viii) the production of an annual report containing evidence-based recommendations to minimize the emergence and transmission of HIVDR. The EWIs, in particular, are attractive for ongoing monitoring because salient data can be retrieved from national ART PMSs [7], at relatively low cost and quicker than the laboratory based assays for the assessment of HIVDR mutations.

A limited number of studies are available from Africa that assesses EWI performance [6,8-11], and none from Tanzania. Furthermore, we could not find any studies that attempt to use programmatic factors as predictors for EWI success. Using four indicators that could be captured using the existing PMS, we undertook a baseline facility-level survey to a) ascertain Tanzanian national performance against WHO targets for four HIVDR EWIs and b) determine the correlates of achievements or failures in reaching the targets.

## Methods

### Overall study design

We conducted a retrospective longitudinal survey in 35 purposively sampled care and treatment clinics (CTCs) of patient cohorts who initiated ART in those facilities in 2010.

### Setting

The Tanzanian health system is comprised of referral hospitals, regional hospitals, district hospitals, health centres and dispensaries. Dispensaries serve as the main point of care, larger health centres provide more complex treatment including inpatient care, and district and regional hospitals are equipped for surgery and referral-level care. HIV and AIDS care and treatment services in Tanzania were approved by the government in October 2003 under the coordination of the National AIDS Control Programme (NACP), and ART roll-out began in October 2004. All facilities provide ART free of charge, and during the time of the study, there were 1,100 facilities nationwide providing ART. The target was to provide ART to 440,000 eligible patients by the end of 2010, but only 244,148 (55%) patients had begun ART by July 2010. During the period of this evaluation, patients were eligible for first-line ART if they were HIV-infected, understood the implications of therapy, and fell into one of these three categories: patients in WHO clinical stage IV, patients in WHO clinical stage III with CD4 count less than 350 cells/dl, or all patients with CD4 count less than 200 cells/dl regardless of clinical criteria [12]. All patients starting first-line ART in Tanzania are registered and followed-up clinically and using laboratory parameters. Normally ART patients attend clinics monthly for drug refills and continued medical check-up. A standardized national patient monitoring system, which is either paper-based or electronic, is in use at all facilities. The reports are produced quarterly through ART and pre-ART registers.

### Sampling

All facilities providing HIV and AIDS care and treatment services were eligible for this survey. We constructed a national sample of facilities from 17 of the 21 regions in mainland Tanzania. We purposefully selected 35 CTCs to represent higher-level facilities (referral or regional hospitals) and lower-level facilities (district hospitals or health centres) from each region to ensure representation of different administrative levels. Our sample included all four referral hospitals, which are distributed across the country’s mainland. In Dar es Salaam Region, we sampled three facilities to account for the density of ART patients. We excluded private facilities because they are few in number, unevenly distributed, and their reporting to the central system has not been well established.

Using the WHO sampling guidance [13], we calculated minimum/finite sample sizes for each EWI, based on number of eligible patients (patients initiated on ART) for each study indicator in 2010, the year prior to the survey. We chose a starting point from a list of charts based on our EWI sample start date (January 1, 2010) and then consecutively sampled charts until we reached minimum sample sizes required to achieve a 95% confidence interval of ± 7% [13]. Health care providers (HCPs) from the sampled facilities conducted the data abstraction for HIVDR EWIs. This approach aimed to build local capacities of facility staffs to be able to assess the programmatic factors associated with HIVDR using their own data. HCPs only included the charts of adult patients (age ≥ 15 years) who initiated ART at their respective facility. They excluded files belonging to patients who transferred in while on ART from another facility after January 2010 in order to avoid double counting had they also been sampled from the facility where they initiated ART. Patients who transferred out (TOs) of the study facilities were eligible for analysis of indicator 1a, regardless of transfer out dates. However, the TOs were excluded from analyses of indicators 2 and 3a only if the transfer out date was during the study period and of indicator 5b if the transfer out date was before the first scheduled appointment after ART initiation.

### Measurement

We gathered survey data using specially designated EWI data abstraction tools. However, our selection of these particular four indicators was based on routinely collected data from NACP’s existing PMS. The four EWIs for which we abstracted data were:

EWI 1a: Percentage of [adult] patients initiating ART at the site during a selected time period who are initially prescribed, or who initially pick up from the pharmacy, an appropriate first-line ART regimen. We determined if patients were initiated on appropriate first-line regimen as per national guidelines and WHO recommendations [12,14].

EWI 2: Percentage of patients initiating ART at the site in a selected time period who are lost to follow-up (LTFU) during the 12 months after starting ART. We defined LTFU as a patient’s failure to attend the clinic for 90 consecutive days (three months) following their last visit, which is NACP’s and WHO’s standard definition.

EWI 3a: Percentage of adult patients initiating ART at the site during a selected time period who are taking an appropriate first-line ART regimen 12 months later. Being on first-line therapy included all mutual drug substitutions and combinations within this regimen category.

EWI 5b: Percentage of patients initiating ART at the site during a selected time period who attended all clinic appointments on time during the first 12 months of ART. In keeping with the WHO definition, we defined missing a clinic appointment as failing to appear on the same day or within seven days of the expected or scheduled clinic consultation date.

Two to three HCPs from each facility received training on nationally validated data abstraction techniques using facility-held care and treatment patient cards (CTC2). The National HIVDR Technical Working Group provided on-site mentorship and supportive supervision during data abstraction to ensure that the tools were filled correctly. Mentors and supervisors verified the data in completed tools. They also administered structured questionnaires to HCPs to collect information on programmatic factors such as facility maturity (number of months providing ART services), average hours clinics are open per day, average time patients spend in the clinic (defined as total time spent at the clinic, cumulatively in all departments, while waiting for and receiving services), and other characteristics in order to understand facility-level determinants of EWI performance.

### Analysis

Based on WHO guidance and sampling methods described in these referenced documents [10,12,14], we calculated that 3,608 charts would need to be abstracted to have enough power to achieve ± 7% precision, 95% of the time. We uploaded the abstracted patient information into WHO HIVResNet excel-based tool [13] to determine the facility-level weighted averages of EWI performance. We assessed national performance for each EWI by comparing the proportion of clinics that met WHO criteria for each indicator. The WHO’s set targets are 100% for EWI 1a, ≤ 20% for EWI 2, ≥ 70% for EWI 3a and ≥ 80% for EWI 5b. We aggregated facility-level data and computed the proportion of facilities that met the WHO EWI targets by various facility characteristics. We analyzed the association between meeting WHO EWIs and facility characteristics using Fisher’s Exact Test in Stata v. 11 (Stata Corporation, College Station, Texas, USA).

### Ethical considerations

The HIVDR monitoring strategy in Tanzania, which includes EWIs, received ethical approval from Tanzania’s National Institute for Medical Research’s (NIMR) Independent Review Board in 2008 (Ref No NIMR/HQ/R.89/Vol. IX.746). The analysis presented in this report is based on secondary analyses of unlinked data; no contact with human subjects occurred.

## Results

We invited 35 facilities to participate in the survey, and all agreed. The 35 facilities included 28 public and 7 faith-based facilities; 4 referral hospitals, 17 non-referral hospitals and 14 health centers; and 20 urban, 6 semi-urban and 9 rural facilities. During 2010, a total of 13,466 adult patients began ART at these facilities; we abstracted data from 3,608 (27%) charts. All facilities adhered to the national ART management guidelines regarding eligibility for ART initiation. Six facilities had been providing ART services for longer than five years while 14 had been in operation for less than three years (Table 1).

**Table 1:** Weighted averages of HIV drug resistance EWI performance from 35 HIV care and treatment facilities, aggregated by programmatic characteristics, Tanzania, 2010

**Programmatic characteristics**			**HIVDR early warning indicators (EWIs)**			
			**EWI 1a, ART prescription practices**	**EWI 2, patients LTFU at 12 months**	**EWI 3a, retention on 1st line ART at 12 months**	**EWI 5b, clinic appointment keeping in first 12 months**
			**WHO target: 100%**	**WHO target: ≤ 20%**	**WHO target: ≥ 70%**	**WHO target: ≥ 80%**
	**Facility counts n (%)**	**Total # initiated on ART in 2010**	**%**	**% (95% CI)**	**% (95% CI)**	**% (95% CI)**
**National average performance on EWIs**	35 (100)	13,466	100	26 (25–26)	54 (53–54)	38 (37–38)
**Facility administrative level**						
Referral hospitals	4 (11)	2346	100	25 (24–26)	53 (52–55)	54 (53–55)
Non-referral hospitals	17 (49)	8512	100	22 (21–23)	56 (55–56)	37 (36–37)
Health centers	14 (40)	2608	100	32 (30–33)	51 (49–52)	31 (30–32)
**Facility geographical locality**						
Urban	20 (57)	11,604	100	22 (22–23)	56 (55–56)	40 (40–41)
Semi-urban	6 (17)	601	100	43 (41–46)	39 (37–41)	29 (28–30)
Rural	9 (26)	1261	100	27 (26–28)	55 (54–56)	33 (32–34)
**Facility ownership**						
Public	28 (80)	9661	100	24 (23–25)	54 (53–55)	34 (33–34)
Faith-based	7 (20)	3805	100	30 (29–31)	53 (52–54)	50 (49–51)
**Facility maturity (years providing ART services)**						
> 5	6 (17)	4684	100	18 (17–19)	61 (60–62)	47 (46–48)
3-5	15 (43)	6174	100	25 (24–26)	52 (51–53)	37 (37–38)
< 3	14 (40)	2608	100	32 (30–33)	51 (49–52)	31 (30–32)
Median: 4 years IQR: 2–5 years						

IQR interquartile range.

EWI 1a, % of adult patients initiating ART at the site who are initially prescribed, or who initially pick up from the pharmacy, an appropriate first-line ART regimen.

EWI 2, % of patients initiating ART at the site who are lost to follow-up (LTFU) 12 months after ART initiation.

EWI 3a, % of adult patients initiating ART at the site who are taking an appropriate first-line ART regimen 12 months later.

EWI 5b, % of patients initiating ART at the site who attended all scheduled or expected clinical consultations on time during the first 12 months.

Of the 3,608 charts we reviewed from sampled facilities, the weighted national average was 100% for EWI 1a, 26% (95% CI 25–26) for EWI 2, 54% (95% CI 53–54) for EWI 3a, and 38% (95% CI 37–38) for EWI 5b (Table 1). Of the 35 clinics, all met the WHO target for EWI 1a, 17 clinics (49%) for EWI 2, 10 clinics (29%) for EWI 3a and 1 clinic (3%) for EWI 5b (Table 2). None of the clinics met all four EWI targets.

**Table 2:** Proportion of ART facilities that meet WHO EWI targets, aggregated by programmatic characteristics, Tanzania, 2010

**Programatic characteristics**		**HIVDR early warning indicators (EWIs)**			
		**EWI 1a, ART prescribing practices**	**EWI 2, patients LTFU at 12 months**	**EWI 3a, retention on 1st line ART at 12 months**	**EWI 5b, clinic appointment keeping in first 12 months**
		**WHO target: 100%**	**WHO target: ≤ 20%**	**WHO target: ≥ 70%**	**WHO target: ≥ 80%**
	**Frequency**	**n (%)**	**n (%)**	**n (%)**	**n (%)**
**National average performance on EWIs**	(N = 35)	35 (100)	17 (49)	10 (29)	1 (3)
**Facility administrative level**					
Referral hospitals	4	4 (100)	2 (50)	0 (0)	1 (25)
Non-referral hospitals	17	17 (100)	7 (41)	5 (29)	0 (0)
Health centers	14	14 (100)	8 (57)	5 (36)	0 (0)
			*p = 0.793*	*p = 0.512*	*p = 0.114*
**Facility locality**					
Urban	20	20 (100)	11 (55)	5 (25)	1 (5)
Semi-urban	6	6 (100)	2 (33)	1 (17)	0 (0)
Rural	9	9 (100)	4 (44)	4 (44)	0 (0)
			*p = 0.727*	*p = 0.582*	*p = 1.000*
**Facility ownership type**					
Public	28	28 (100)	13 (46)	8 (29)	0 (0)
Faith-based	7	7 (100)	4 (57)	2 (29)	1 (14)
			*p = 0.691*	*p = 1.000*	*p = 0.200*
**Facility maturity (years providing ART services)**					
> 5	6	6 (100)	4 (67)	2 (33)	1 (17)
3-5	15	15 (100)	5 (33)	3 (20)	0 (0)
< 3	14	14 (100)	8 (57)	5 (36)	0 (0)
			*p = 0.324*	*p = 0.611*	*p = 0.171*

EWI 1a, % of adult patients initiating ART at the site who are initially prescribed, or who initially pick up from the pharmacy, an appropriate first-line ART regimen.

EWI 2, % of patients initiating ART at the site who are lost to follow-up (LTFU) 12 months after ART initiation.

EWI 3a, % of adult patients initiating ART at the site who are taking an appropriate first-line ART regimen 12 months later.

EWI 5b, % of patients initiating ART at the site who attended all scheduled or expected clinical consultations on time during the first 12 months.

Twenty-nine (83%) ART facilities were operating their clinics for at least eight hours per day. Patients at 6 (17%) facilities travelled an average of less than 10 kilometers, whereas those at 16 (46%) facilities travelled over 30 kilometers on average to attend the clinic. At 14 (40%) facilities the average time patients spent in the clinic (which included wait time and time spent with providers) was less than 60 minutes, and at 6 (17%) it was over 180 minutes. Thirty (86%) facilities had no supplementary ART dispensing locations; only two (6%) ART facilities provided both refill sites and outreach services for ART. Twenty-five (71%) facilities provided home-based services.

Facilities that met EWI 2 and 3a were more likely to have their patients spend more than 180 minutes on average in the clinic (p = 0.011 and 0.007, respectively), Table 3. The only facility that met EWI 5b was a faith-based urban referral hospital operating for less than 8 hours per day open.

**Table 3:** Proportion of ART facilities that met WHO EWI targets, aggregated by additional programmatic/ facility characteristics, Tanzania, 2010

		**EWI 1a, ART prescribing practices**	**EWI 2, patients LTFU at 12 months**	**EWI 3a, retention on 1st line ART at 12 months**	**EWI 5b, Clinic appointment keeping in first 12 months**
		**WHO target: 100%**	**WHO target: ≤ 20%**	**WHO target: ≥ 70%**	**WHO target: ≥ 80%**
	**Frequency**	**n (%)**	**n (%)**	**n (%)**	**n (%)**
**Average opening time (hours)**					
Clinic & pharmacy: ≥ 8 hrs	29	29 (100)	16 (55)	10 (35)	0 (0)
Clinic & pharmacy: < 8 hrs	6	6 (100)	1 (17)	0 (0)	1 (17)
			*p = 0.177*	*p = 0.640*	*p = 0.171*
**Average distance (kilometers, km) travelled by patient**					
≥ 51 km	10	10 (100)	5 (50)	3 (30)	1 (10)
21-50 km	10	10 (100)	5 (50)	3 (30)	0 (0)
11-20 km	8	8 (100)	6 (75)	3 (38)	0 (0)
≤ 10 km	7	7 (100)	1 (14)	1 (14)	0 (0)
			*p = 0.162*	*p = 0.885*	*p = 1.000*
**Average time (minutes) patient spent at the Clinic**					
>180 minutes	6	6 (100)	6 (100)	5 (83)	0 (0)
60-180 minutes	15	15 (100)	7 (47)	3 (20)	1 (7)
< 60 minutes	14	14 (100)	4 (29)	2 (14)	0 (0)
			p = 0.011*	p = 0.007*	*p = 1.000*
**Supplementary ART dispensing locations**					
Refill, outreach or both	5	5 (100)	2 (40)	2 (40)	0 (0)
Neither	30	30 (100)	15 (50)	8 (27)	1 (3)
			*p = 1.000*	*p = 0.610*	*p = 1.000*
**Home based services**					
Yes	25	25 (100)	13 (52)	6 (24)	1 (4)
No	10	10 (100)	4 (40)	4 (40)	0 (0)
			*p = 0.711*	*p = 0.421*	*p = 1.000*

*Statistically significant.

EWI 1a, % of adult patients initiating ART at the site* who are initially prescribed, or who initially pick up from the pharmacy, an appropriate first-line ART regimen.

EWI 2, % of patients initiating ART at the site* who are lost to follow-up (LTFU) 12 months after ART initiation.

EWI 3a, % of adult patients initiating ART at the site* who are taking an appropriate first-line ART regimen 12 months later.

EWI 5b, % of patients initiating ART at the site* who attended all scheduled or expected clinical consultations on time during the first 12 months.

## Discussion

We found that all surveyed facilities had 100% of patients initiated on appropriate first-line ART regimens (EWI 1a). Meeting EWI 1a is a critical success because initiating patients on a mixture of therapies would a) complicate initiation protocols and require routine genotyping at baseline, which is not currently viable in Tanzania; b) tap into usable therapies that should be conserved for patients who fail first-line treatment; c) preclude the system from monitoring patients who fail first-line and must be switched to second-line regimens, a crucial indicator for detecting resistance.

On the other hand, we found that none of the facilities surveyed met all four WHO EWI targets, and only one facility met EWI 5b (ART clinic appointment keeping during the first 12 months of ART). These results are very concerning, as missing even a few doses - particularly during the first six months of therapy when viral loads are high - can lead to treatment failure and drug resistance [2,15,16]. A WHO report found that among 27 African countries surveyed, less than half of the clinics met this target, and globally, less than three fifths of the clinics met EWI 5b [5].

Anecdotally, pharmacy record keeping in Tanzania is inconsistent, therefore missing appointments is the next best proxy for estimating treatment interruption [5]. It is possible, however, that some patients are missing ART clinic appointments but still picking up their antiretrovirals at the pharmacy, so we cannot conclusively say that treatment interruption is occurring at this magnitude. Nevertheless, the results for EWI 5b are alarming. Furthermore, WHO's recently harmonized set of early warning indicators no longer includes EWI 5 [17], which means appointment keeping will no longer be a proxy for ART adherence and possible drug failure.

We also found that facilities whose patients spent the most time in the clinic (more than 3 hours, cumulatively in all departments *including* wait time), were more likely (without controlling for clinics’ ART cohorts sizes) to fulfill EWI 2 (LTFU 12 months after ART initiation) and EWI 3a (retention on first-line ART at 12 months) than others. Since we are not able to distinguish wait time from time spent with providers, we have two possible explanations. If longer time spent in the clinic is, in fact, due to more patient-provider contact time, there is a sizeable body of literature on patient satisfaction relative to time spent with provider [18,19], and we speculate that greater patient satisfaction may lead to lower rates of LTFU. We also theorize that patients who spend more time with their providers may understand and adhere to their regimen better, leading to improved retention on first-line regimens. Conversely, if longer visits were due to greater wait times, research shows that patient satisfaction may be high even when wait times are long [20,21]. Furthermore, it is plausible that the association between longer wait times and higher EWI performance is confounded by higher quality services at some facilities.

Hypothetically, patients may be willing to wait longer at facilities they consider higher quality (with more skilled providers and better diagnostic equipment), while these facilities are also more likely to perform higher in EWIs. Nonetheless, the association between time spent in the clinic and performance in EWI 2 and 3a should be interpreted with caution, as measurement error and respondent bias may be a factor. If providers overestimated the average time they spend with patients, we would expect to see a smaller effect size after taking this into account.

We were not successful in identifying many significant predictors of high EWI performance. Referral hospitals did not perform better than lower level facilities with regard to patient retention on first-line ART (EWI 3a) as we expected. Perhaps this is because more experienced clinicians in referral hospitals may be better equipped to recognize patients who are failing their first-line regimen and move them to second-line therapy. We also expected that facilities with supplemental ART dispensing locations (such as refill sites and outreach services) and home based care would be more successful with LTFU and appointment keeping, but this was not the case. It is possible that with a few sampled facilities and only some facilities providing outreach services, that we did not have sufficient power to detect this difference. Lastly, we anticipated facilities whose patients walk longer average distances would perform worse overall, but perhaps some patients choose to travel to more distal facilities to avoid the stigma of receiving treatment near their homes.

Our study is subject to several limitations. It is the first assessment of its kind undertaken in Tanzania, and in future studies, we hope to obtain better measures for treatment interruption, as well as more robust measures of facility characteristics, such as patient visit times, to better understand how patients spending more time in the clinic results in less attrition and greater retention on first-line ART. Given the human resource shortages Tanzania faces, we are not suggesting that providers should spend more time with patients, but that understanding the factors that contribute to more efficient clinic visits is crucial for balancing human resource constraints with providing both effective and quality care. Secondly, our sample was not perfectly representative of all ART facilities in the nation, and we plan to extend subsequent surveys to areas not covered in this initial survey, including the private facilities, as well as to randomly sample lower-level facilities to avoid selection bias. Finally, there are inherent limitations in the survey methods we employed. We were unable to develop data on the WHO HIVDR EWIs that cannot be obtained from routine patient monitoring, which we plan to address with additional special surveys in our next monitoring round. Additionally, we plan in the future to triangulate EWI data, like those collected in this study, with HIVDR threshold and monitoring surveys, both of which are undergoing development. Despite these limitations, we believe that our data point out successes (initiating ART) and where our system needs immediate improvements (attrition, retention on first-line ART and clinic appointment keeping). It is with periodic surveys such as this that ART services in Tanzania can be improved to provide the best care and treatment for our patients.

## Conclusion

In conclusion, Tanzania performed very well in EWI 1a, ART prescribing practices, but its performance in the other three EWIs fell far short of the WHO targets. This study provides a baseline for future monitoring of EWIs in Tanzania. It also conveys lessons learned for surveys that aim to identify practices contributing to the emergence or prevention of HIVDR. EWIs are a quicker assessment technique and they are crucial for tracking HIVDR in resource-limited settings where individual-level monitoring is not feasible. In addition to the national monitoring of EWIs, clinics should be empowered to use their own data to track EWIs more routinely at a facility level in order to address problems or gaps in a timely manner. With better understanding of the programmatic factors that contribute to decreased HIV drug resistance, we may continue to improve the quality of ART services provided for Tanzanians living with HIV and AIDS, and ultimately, the quality of their lives.

## Abbreviations

AIDS: Acquired immunodeficiency syndrome; ART: Antiretroviral therapy; CTCs: Care and treatment clinics; EWI: Early warning indicator; HIV: Human immunodeficiency virus; HIVDR: HIV drug resistance HCPs - health care providers; LTFU: Lost to follow up; NACP: National AIDS control programme; NIMR: National institute for medical research; PMS: Patient monitoring system; WHO: World Health Organization.

## Competing interests

The authors declare that they have no competing interests.

## Authors’ contributions

JMJ Tools design, survey field work, preliminary data aggregation and local feedback report compilation, secondary data analysis and primary and final manuscript write-up and approval. TJ, AM secondary data analysis and interpretation, and primary and final manuscript write up and approval. SGR, RAA, SV, BR, KB, JSB Tools design, Survey field work, preliminary data aggregation and Local feedback report compilation, final manuscript write-up and approval.

## Pre-publication history

The pre-publication history for this paper can be accessed here:

http://www.biomedcentral.com/1471-2334/14/196/prepub
